# With Appreciation

**DOI:** 10.1016/j.cdnut.2023.100037

**Published:** 2023-02-15

We thank the following individuals for taking the time necessary to evaluate original manuscripts. Anonymous, conscientious, fair, and timely peer review is the lifeblood of the journal.

**Table:** 

Samiaa Jamil Abdulwahid
Frances Aboud
Yubraj Acharya
O.Yaw Addo
Olasunkanmi Adegoke
Prekshi Aggarwal
Noha Ahmed Nasef
Bridget Augusta Aidam
Kolapo Ajuwon
Diana Allotey
Alex K. Anderson
Andres Ardisson Korat
Gaston Ares
Aweke Kebede Arfassa
Mary Arimond
Seth Armah
Patrice Armstrong
Richmond Aryeetey

Mara Baller
Kathryn Barger
Daniela Barile
Megan R. Beggs
Rhonda Bell
Jon Bennett
Julia K. Bird
Jackie Boerman
Sarah L. Booth
Carol Boushey
Shanthy Bowman
Didier Brassard
Judith Brown
Andrew W. Brown
Richard Bruno

Mariana Calle
Eric Calloway
Mary Ellen Camire
Theresa Casey
Shanon L. Casperson
Chimene Castor
Devon Cataldi
Anindita Ray Chakravarti
Katherine Chen
Sabiha Sultana Chowdhury
Amina Chughtai
Soonkyu Chung
Ilana Rachel Cliffer
Alison M. Coates
Shalean Collins
David Colozza
Joanna Cummings

Shaonong Dang
Omar Dary
Hassan S. Dashti
F.J. de Gooijer
Jan de Vries
Radhouene Doggui
Carmen Marino Donangelo
Shauna M. Downs
Caitlin Dreisbach

Andrea Elliot
Dolapo Enahoro
John Erdman
Temitope Erinosho

Lindy Fenlason
Rafaela Feresin
Bradley Ferguson
Barbara Fiese
Amelia B. Finaret
John Finley
Cindy E. Fitch
Catharine A.K. Fleming
Maryah Fram
Cara Frankenfeld
Valerie M. Friesen
Edward A. Frongillo

Lena Galvez Ranilla
Xiang Gao
Sappho Gilbert
Nana Gletsu-Miller
Christopher Golden
Jessica A. Grieger
Craig Gundersen

Reza Hakkak
Haewook Han
Jacob M. Haus
Siran He
Rebecca Heidkamp
Jaimie Hemsworth
Kirsten Herrick
Julie M. Hess
Sonja Y. Hess
Kenneth Ho
Rebecca A. Hodge
Jessie Baldwin Hoffman
Vivian Hoffmann
Emily E. Hohman
Tori Holthaus
Amber Hromi-Fiedler
Van S. Hubbard
Sheryl O. Hughes
Dana Hunnes
Richard Hurrell

Scott Ickes
Fumiaki Imamura
Amy Ing
Deen Islam

Julie Jesson
Qing Jiang
Rulan Jiang
Xinyin Jiang

Mary E. Kable
Kelly Kane
Mitch Kanter
Justine A. Kavle
Melissa Cunningham Kay
Michael J. Keenan
Julie Kennel
Naiman Khan

Matthew J. Landry
Anna Lartey
Marlou Lasschuijt
Anne Sofie Dam Laursen
Jennifer Lee
Lambert Leong
Cheng Li
Brian L. Lindshield
Xiaoran Liu
WaiYee Low
Jing Lu
Bret Luick
Hanqi Luo
Chessa Lutter

Margaret Marmar-Klemesu
Stephanie L. Martin
Andressa Martins
Mónica Mazariegos
Megan A. McCrory
Alexander McLain
Sarah McNaughton
Rukshan Mehta
Tagesse Abo Melketo
David Miller
Erin M. Milner
Edwin Miranda
Kris M. Mogensen
Diana Montoya-Williams
Kelly Moore
Nancy E. Moran
Regis Moreau
Juliano Morimoto
Tamara Yousef Mousa
Mercy Mwambi
Martha Kaeni Mwangome

Sarah Nájera Espinosa
Paulo A.R. Neves
Emmanuel Nshakira Rukundo

Brietta M. Oaks
Lauren Elizabeth O'Connor
Marie-Claire O'Shea
Bridget Owens
Ibukun Owoputi

Ryan M. Pace
Aunchalee Palmquist
Bunga Astria Paramashanti
Ben Parmenter
Simone Passarelli
Giulia Pastori
Rafael Perez-Escamilla
Kristina S. Petersen
Stuart Phillips
Yan Yin Phoi
Sara Police
Rebecca Pradeilles

Dima Qato

Amanda Raffoul
Kalyani Raghunathan
Ashfikur Rahman
Elizabeth Rhodes
Eduardo Rico
Malcolm D. Riley
Christian Ritz
Julian Robbins
Nancy Rodriguez
Arpita Roy
Julie Ruel-Bergeron
Jill Ryer-Powder

Saikrishna S
Edward Saltzman
Flavia Mori Sarti
Samuel Scott
Paraskevi Seferidi
David S. Seres
Andrew Shao
Amrollah Sharifi
Indu Kumari Sharma
Sangita Sharma
M. Kyla Shea
John Shepherd
Kalidas Shetty
Sonya S. Shin
Richard Siegel
Jesus Simal-Gandara
Joanne Slavin
Brenda Smith
Elliot O'Brian Smith
Barbara Stoecker
Sarah Stotz
Rita Strakovsky
April Stull
Karen Switkowski

Elad Tako
Elise Talsma
Gourab Raj Talukdar
Libo Tan
Christopher A. Taylor
Sarah Taylor
Jeffery Sivert Tessem
Emily J. Tomayko
Liv Elin Torheim
Samantha Torres
Armando R. Tovar
Claire Nora Tugault-Lafleur

Maya K. Vadiveloo
Dustin Valdez
Marie van der Merwe
Krista Varady
Tonia Vassilakou
Sonia Vega-López
Amanda Vest
Florent Vieux
Colby Vorland

Karen Walton
Shu Wang
William Waters
Jingkai Wei
Michael Wenger
Lynne Wilkens
Anne M. Williams
Mary Williams
Pattanee Winichagoon
Thomas Wolfgruber
Michael Wong
Di Wu

Zhenyu Yang
Bridget Young

Yiying Zhao
Jean-Marc Zingg
Christina Zorbas

